# 

**Published:** 1921-05-14

**Authors:** 


### Passing Events and Topics.

### From Weekly to Fortnightly.

The *New York Medical Journal*, established in 1843, has from its earliest days proved to be one of the most useful and reliable of the weekly American medical journals. With it was incorporated in 1903 the *Philadelphia Medical Journal*, and in 1905 the *Medical News*, also founded in 1843. Thus three well-known medical journals were combined. Now another change has to be recorded in the history of this journal, for from March 2, 1921, it will be published on the first and third Wednesdays in each month instead of weekly. Its present production, with illustrations, retains its high character, but it now carries a yellow cover.

### East Cowes Hospital Extension.

The demands on the Frank James Memorial Cottage Hospital, East Cowes, have been so great that the old operating-theatre has been converted into a men's ward and a new theatre provided, together with all the usual annexes and a dispensary.

### To Help the London Temperance Hospital.

A most successful entertainment by the nursing staff of the London Temperance Hospital was given on a recent evening in the large out-patient hall of the hospital, before a full and most appreciative audience. The crowded programme of musical items and amateur theatricals was carried through without a hitch or a dull moment, and with the enthusiasm that makes for success. Prices of admission varied from 10s. 6d. to 1s., and the proceeds were for the benefit of the building fund to reconstruct the Nurses' Home and open another ward of twenty beds. The entertainment was repeated on two succeeding evenings.

### Memorial to a Leicester Physician.

A memorial to the late Dr. T. Arnold Johnston, assistant physician and pathologist to the Leicester Royal Infirmary, who died on February 4, 1918, was unveiled by Mr. N. C. Ridley, F.R.C.S. (ophthalmic surgeon), the senior member of the honorary medical and surgical staff. The tablet commemorates a life freely given in the sacred cause of humanity and at the call of duty, fighting a subtle and insidious foe in the laboratory. It will show to future generations that the Leicester Royal Infirmary appreciated her sons and lamented a young and promising life cut untimely short.

### Army Schools of Hygiene.

Permanent centres of instruction for staff and regimental officers and for regimental sanitary *personnel* in the principles on which hygiene is based are being established at Aldershot (when the transfer of the school at Blackpool is effected); Netley, Hants; Strensall Camp, near York; and Carrickfergus.

### Tablets for Voluntary Hospitals.

The County of London Branch of the British Red Cross Society has suggested that the London County Council should affix tablets on buildings used as military hospitals in the war. The Council cannot see their way to incur this expenditure, and has suggested that the Red Cross Society and Order of St. John should undertake the work.

### Matrons' and Sisters' Appointments.

Chester Royal Infirmary.—Miss E. Richards has been appointed theatre sister. She was trained at the Royal Infirmary, Sheffield, where she was later theatre assistant.

Blair Convalescent Hospital, Bromley Cross, Bolton.—Miss Alice H. Scott has been appointed sister. She was trained at Brownlow Hill Infirmary, Liverpool, and has been sister at the Wilkinson Sanatorium, Bolton, at the Royal Naval Hospital, Dungavel, N.B., night superintendent at Middleton-in-Wharfedale Sanatorium, and senior sister at Accrington Victoria Hospital. She is a member of the College of Nursing.

Harton Hospital, North Shields.—Miss J. MacRonley has been appointed ward sister. She was trained at Newcastle Union Infirmary, and has been health visitor for Tynemouth Corporation.

Maternity Hospital, Bromley.—Miss Ellen Lauder, A.R.R.C., has been appointed matron. She was trained at the Middlesex and Queen Charlotte's Hospitals, and was sister-in-charge of the in- and out-patients' department at the former. She served in the T.F.N.S for five years as assistant matron.

Bethnal Green Hospital.—Miss Bessie Slater has been appointed theatre sister. She was trained at the London Hospital, was on active service as sister for three years during the war, and then theatre sister under the British Red Cross.

Basford Union Infirmary.—Miss E. E. Healey has been appointed superintendent nurse. She was trained at Selly Oak Infirmary, has been matron of Winchcombe Poor Law Institution, charge nurse at Maidstone Poor Law Infirmary, and superintendent nurse of Whitehaven Poor Law Infirmary.

Middleton-in-Wharfedale Sanatorium.—Miss M. F. Stockwell has been appointed home sister. She was trained at University College Hospital, and has been sister at Queen Mary's Hospital, Stratford, and at the Jessop Hospital, Sheffield. She served in France for four years.

St. Leonard's Hospital, Sudbury.—Miss L. I. Everitt has been appointed sister. She was trained at St. Marylebone Infirmary, where she has been theatre nurse. She has done private and district nursing, and served with Q.A.I.M.N.S.R. in Salonica and at home as sister and night superintendent. She is a member of the College of Nursing and holds the C.M.B. certificate.

Eryri Hospital, Carnarvon.—Miss Martha Williams has been appointed head nurse. She was trained at Kensington Union Infirmary, was district nurse at Tottenham, staff nurse at the New End Military Hospital, served in Q.A.I.M.N.S. at Connaught Hospital, Aldershot, and at present is sister-in-charge of a ward at the Hackney Union Infirmary.

Great Northern Central Hospital's Recovery Home, Southgate.—Miss Beatrice J. Everingham has been appointed matron. She was trained at the Great Northern Central Hospital, where she was later sister; she served with the T.F.N.S. for five years, and returned as sister to her training-school.

Wellhouse Hospital, Barnet.—Miss Fanny Fairbairn and Miss Maude Mallett have been appointed ward sisters. Miss Fairbairn was trained at the Royal Victoria Hospital, Folkestone, and has been staff nurse at the Grove Military Hospital and holiday sister at St. James's Infirmary, Balham. Miss Mallett was trained at Hampstead General Hospital, and has been temporary sister at the Farnham Infirmary. She has also done private nursing.

### Echo of the Welsh Railway Accident.

The directors of the Cambrian Railway Co. have shown their appreciation of the services rendered by the Montgomery County Infirmary at the disastrous railway accident at Abermule by a donation of 200 guineas towards the funds of the institution. The windfall has come at a most opportune time, as there was a serious deficit on the year's expenditure. The services of the matron and staff have also been very kindly acknowledged, whilst a further memorial of the disaster is a handsome clock, presented to the women's ward by the Llandrinan Free Church Women's Guild in memory of Nurse Gethin, of that parish, who lost her life in the accident when returning to her duties at the London Temperance Hospital.

May 14, 1921] [Price Twopence

### The Hospital

**RATES OF SUBSCRIPTION** (post free, payable in advance).

Three months, 3/3; six months, 6/6; twelve months, 13/-. (Should the cost of printing and paper as well as increased postage, necessitate alteration of these rates, the period paid for will be reduced proportionately on unexpired subscriptions.) THE HOSPITAL "Index" to Volume LXIX., October 1920 to March 1921, is now ready, price 1/1 per copy, post free. Address The Manager, THE HOSPITAL, "The Hospital" Building, 28/29 Southampton Street, Strand, London, W.C. 2.

